# A Cross-Sectional Prospective Study of Cutaneous Lesions in Newborn

**DOI:** 10.1155/2014/360590

**Published:** 2014-01-20

**Authors:** Farhana Tahseen Taj Sameer Haveri, Arun C. Inamadar

**Affiliations:** ^1^Department of Dermatology, Dr. Prabhakar Kore Hospital and Medical Research Centre, KLE University's JN Medical College, H. No. 2, Second Cross, Veerbhadra Nagar, Belgaum, Karnataka 590010, India; ^2^Department of Dermatology, Shri B. M. Patil Medical College, BLDE University, Bijapur, Karnataka 586103, India

## Abstract

*Background*. Cutaneous alterations are common in neonates. The majority of lesions are physiological, transient, or self-limited and require no therapy. Although much has been reported on the various disorders peculiar to the skin of infant, very little is known about variations and activity of the skin in neonates. *Objective*. To study the various pattern of skin lesions in newborn and to estimate the prevalence of physiological and pathological skin lesions in newborn. *Methods*. A total of 1000 newborns were examined in a hospital-based, cross-sectional prospective study in the period of November 2007 to May 2009. *Results*. The physiological skin changes observed in order of frequency were sebaceous gland hyperplasia (89.4%), Epstein pearls (89.1%), Mongolian spot (84.7%), knuckle pigmentation (57.9%), linea nigra (44.5%), hypertrichosis (35.3%), miniature puberty (13.3%), acrocyanosis (30.9%), physiological scaling (10.8%), and vernix caseosa (7.7%). Of the transient noninfective conditions, erythema toxicum neonatorum was seen in 23.2% newborns and miliaria crystallina in 3% newborns. The birthmarks in descending order of frequency were salmon patch (20.7%), congenital melanocytic nevi (1.9%), and café-au-lait macule (1.3%). Cutaneous signs of spinal dysraphism were sacral dimple (12.8%), meningomyelocele (0.5%), acrochordons (0.1%), and dermoid cyst (0.1%). *Conclusion*. The physiological and transient skin lesions are common in newborns particularly sebaceous gland hyperplasia, Epstein pearls, Mongolian spots, and erythema toxicum neonatorum. It is important to differentiate them from other more serious skin conditions to avoid unnecessary therapeutic interventions.

## 1. Introduction

The newborn or neonatal period is the first 4 weeks of extrauterine life. The skin of the neonate differs from adult in several ways. The thickness of newborn skin is 40% to 60% of that of adult skin. It has weaker intercellular attachment and produces lesser amount of sweat.

A host of aberrations varying from physiological (Mongolian spot) and transient (erythema toxicum neonatorum) to grossly pathological (neonatal lupus erythematosus) are seen in the skin of neonates.

Majority of the neonatal cutaneous lesions are physiological and transient requiring no therapy. However, these cause concern not only to the parents but also to the physicians who are unfamiliar with these skin changes in newborn.

It is necessary to differentiate between benign and clinically significant skin lesions in newborn. Pigmented lesions at birth, such as Mongolian spots, are benign and almost always disappear by few years, whereas congenital melanocytic nevi are clinically significant because of future risk of malignant melanoma.

Therefore it is important to be aware of the innocent transient skin lesions in newborn and differentiate these from other serious conditions which will help avoid unnecessary therapy to the neonates and the parents can be assured of good prognosis of these skin manifestations.

The neonatal skin changes show a wide geographic and ethnic variation. Some skin lesions are common in darker skin races and *vice versa. *It is important to know the pattern of dermatoses prevalent among Indian children at the neonatal period. However, studies on neonatal dermatoses, conducted in India, are limited. Hence, this study has been planned to know the prevalence of different cutaneous lesions among newborns in India.

## 2. Methodology

### 2.1. Source of Data

A hospital-based, cross-sectional, prospective study was conducted in the Department of Dermatology, Venereology and Leprosy, BLDE University, Shri B M Patil Medical College Hospital and Research Centre, Bijapur. One thousand neonates delivered in the Department of Obstetrics and Gynaecology of the same institution were surveyed for the presence of skin lesions. The study was conducted in the period of November 2007 to May 2009.

### 2.2. Method of Collocation of Data

#### 2.2.1. Inclusion Criteria

Neonates within the first 4 weeks of life irrespective of gestational age, sex, and mode of delivery were included in the study.

#### 2.2.2. Exclusion Criteria

Neonates kept in neonatal intensive care unit were excluded from the study, as repeated handling of the babies can increase the chances of infection and sepsis.

#### 2.2.3. Procedure

Detailed history was recorded especially age of the mother, parity of mother, history of consanguinity, mode of delivery, and history of maternal illness during pregnancy. The neonates were examined thoroughly in daylight with accurate definition of morphology of skin lesions and findings were recorded. The sex, birth weight and age at the time of examination were noted in each case. In all instances, diagnosis of disorder was based on clinical impression. Skin biopsy was done in one case.

#### 2.2.4. Statistical Analysis

The observations pertaining to parameters under study among the newborn babies are expressed in percentage.

The relationship between skin lesions and various maternal-neonatal aspects is calculated using *Z*-test, with *P* ≤ 0.05 considered statistically significant.

## 3. Results

Among 1000 newborns, 543 (54.3%) were males and 457 (45.7%) were females. Term newborns were 891 (89%), preterm were 93 (9.3%), and postterm were 16 (1.6%). Five seventy five (57.5%) newborns weighed <2.50 kg and 424 (42.4%) weighed >2.50 kg. History of consanguinity was present in 442 (44.2%) and absent in 558 (55.8%). The route of delivery was normal vaginal route in 688 (68.8%) and caesarean section in 312 (31.2%). The maximum number of mothers as in age group 20–30 (83.7%), 133 (13.3%) were of <20 yrs of age, and 30 (3.0%) were in age group >30–35 yrs. The relationship of skin lesions with maternal and neonatal factors is given in Table 1. The frequency of skin lesions in newborns is given in Table 2. Of all the cutaneous lesions in the newborn, physiological skin lesions were more common in 5911 (59.1%), followed by transient noninfective conditions in 263 (26.3%), eczematous eruptions in 13 (1.3%), birthmarks in 241 (24.1%), cutaneous signs of spinal dysraphism in 135 (13.5%), and others in 25 (2.5%).

Among physiological skin lesions, sebaceous gland hyperplasia (Figure 2) was most commonly seen in 894 (89.4%) neonates, among which 428 (47.87%) were females and 466 (52.12%) were males. The most common site of location was nose. Epstein pearls were seen in 891 (89.1%) newborns with 399 (44.78%) females and 492 (55.21%) males. The most common site of location was midline of palate; it was seen over gingiva (Figure 6) in 10 (1.12%) newborns. Mongolian spots (Figure 9) were seen in 847 (84.7%) newborns: 380 (44.86%) females and 467 (55.13%) males; most common site of location was lumbosacral area. It was multiple in 47 (5.54%) newborns. Milia (Figure 4) was seen in 18.3% newborns.

**Figure 1:**
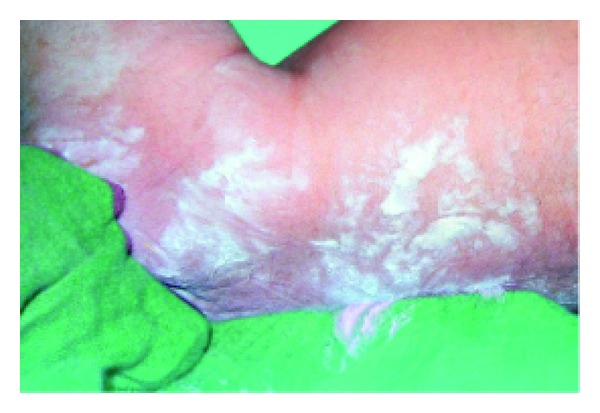
Vernix caseosa.

**Figure 2:**
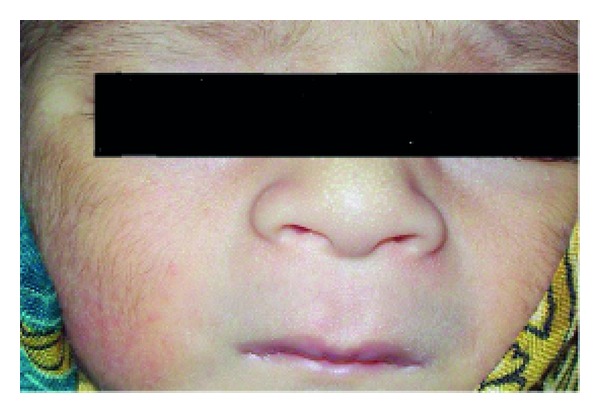
Sebaceous gland hyperplasia.

There was no statistically significant difference in distribution of physiological skin lesions among males and females except for few cutaneous manifestations like vernix caseosa, physiological scaling, Epstein pearls, hypertrichosis, linea nigra, genital pigmentation, pigmentation of pinna, knuckle pigmentation, Mongolian spot, and acrocyanosis. As shown in Table 3, vernix caseosa (Figure 1) was seen commonly in females compared to males with a statistically significant (*P* < 0.05) difference, whereas all other physiological skin lesions were seen commonly in males.

**Figure 3:**
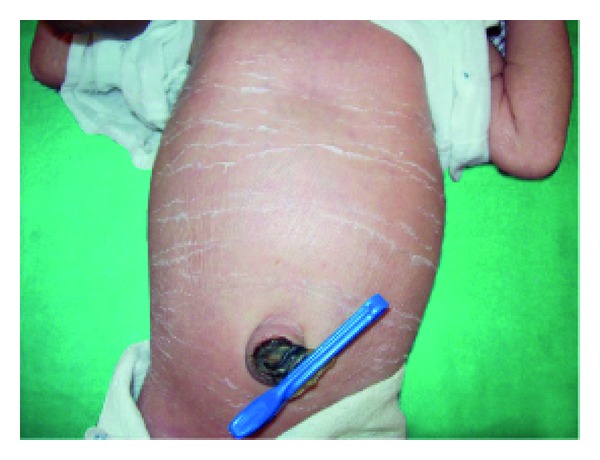
Physiological scaling of newborn.

**Figure 4:**
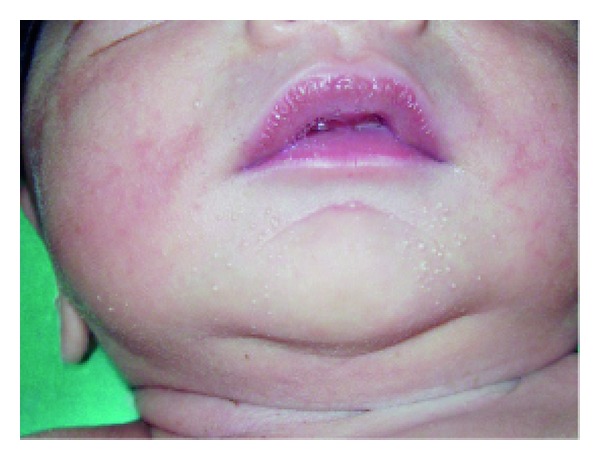
Milia.

With respect to maturity, all skin lesions were commonly seen in term newborns, compared to preterm and postterm newborns. Vernix caseosa was seen in 62 (92.8%) full-term, 4 (5.9%) preterm, and 1 (1.5%) postterm neonates. Physiological scaling (Figure 3) was seen in 97 (92.38%) full-term, 5 (4.76%) preterm, and 3 (2.88%) postterm neonates. Distribution of skin lesions with respect to maturity is given in Table 4. Erythema toxicum neonatorum (Figure 10) was the common transient noninfective condition seen in 232 (23.2%) newborns, followed by miliaria crystallina (Figure 11) seen in 30 (3.0%) newborns and eosinophilic pustulosis in 1 (0.1%). Erythema toxicum neonatorum was seen in 107 (46.3%) females and 125 (53.37%) males and this difference was not statistically significant (*P* = 0.0947). It was seen commonly in 218 full-term (93.96%), 12 (15.17%) preterm, and 2 (0.87%) postterm neonates.

Miliaria crystallina was seen in 20 (66.67%) males and 10 (33.33%) females and this difference was statistically significant (*P* = 0.0098). It was seen in 23 (76.67%) full-term neonates, 6 (20%) preterm neonates, and 1 (3.33%) postterm neonate. Eczematous eruption was seen in 14 neonates, cradle cap in 13 (1.3%) newborns, and napkin dermatitis in 1 preterm neonate.

Vascular birthmarks were seen in 209 (20.9%) and pigmentary birthmarks in 32 (3.2%). Salmon patch (Figure 13) was seen in 207 (20.7%), haemangioma in 2 (0.2%) neonates, congenital melanocytic nevi in 19 (1.9%), and café-au-lait macules (Figure 12) in 13 (1.3%) neonates. Distributions of birthmarks in neonates are represented in Table 5. Salmon patch was seen in 101 (48.79%) females and 106 (51.21%) males with no statistically significant difference (*P* = 0.06231). Most common location was eyelids 188 (90.82%) followed by forehead 11 (8.31%) and nape of neck 8 (3.87%).

Congenital melanocytic nevi were seen in 19 (1.9%) newborns, out of which 11 (57.89%) were males and 8 were (42.11%) females (*P* = 0.3304). All had single lesion, with size <1.5 cm.

Café-au-lait macule was seen in 13 (1.3%) newborns, out of which 7 (53.85%) were females and 6 (46.15%) were males (*P* = 0.5365). All had single café-au-lait macule except one who had multiple café-au-lait macules, and similar lesions were also seen in her mother and sibling.

Cutaneous signs of spinal dysraphism were seen in 135 (13.5%) newborns. Sacral dimple was most commonly seen in 128 (12.8%) neonates, meningomyelocele in 5 (0.5%), dermoid cyst in 1 (0.1%), and acrochordons in 1 (0.1%) neonate.

Other developmental defects seen were cleft lip (0.1%), cleft lip and palate (0.1%), supernumerary nipple (0.1%), umbilical granuloma (0.1%), adnexal polyp (0.1%), and accessory tragus (0.1%).

Anhidrotic ectodermal dysplasia (Figures 7 and 8) was seen in 1 (0.1%) neonate, where skin biopsy showed absence of sweat glands. Vaginal tags were seen in 18 (1.8%) newborns and cowlicks hair pattern in 2 (0.2%) neonates. One newborn was HIV positive (0.1%) and there was history of maternal varicella in 2 (0.2%) neonates. Twenty (0.2%) newborns were outcome of twin pregnancy in this study.

## 4. Discussion

The appreciation of normal phenomena and their differentiation from the more significant cutaneous disorders of the neonate is critical. The prevalence of dermatoses among newborns has been documented in various studies conducted in different racial groups (Table 6).

Sebaceous gland hyperplasia (SGH), Epstein pearls (EP), Mongolian spot (MS), and erythema toxicum neonatorum (ETN) are the skin lesions which were commonly seen in the study. The prevalence of skin lesions is comparable to that of the previous study results [1–4] except sebaceous gland hyperplasia which has shown the highest prevalence (89.4%) in the present study. It was seen commonly in term neonates 789 (88.25%). Sebum secretion rates are high in neonates compared with preadolescent children. It is assumed that this sebaceous gland activity reflects the stimulation by placentally transferred maternal androgen, particularly by dehydroepiandrosterone [5].

Mongolian spot has been shown to be a good example of interracial difference. The prevalence of Mongolian spot has been as high as 80 to 90% in Asians [6, 7], and it has been as low as 3 to 10% in Caucasians [4, 8]. In Indians, the prevalence varies from 72 to 89% [6, 9–11]. In the present study, 84.7% of newborns had this birthmark, similar to that of the study conducted by Dash et al. [6].

Epstein pearls were seen in 891 (89.1%) neonates, with the commonest site of location being midline of palate. They occur commonly in 64–89% of normal neonates and are common in Caucasian infants. The similar prevalence rate has been noted in an Indian study conducted by Nanda et al. [9].

Erythema toxicum neonatorum was seen in 23.2% of neonates, similar to previous study conducted in India [9–11]. It was seen within 48 hrs of life, most commonly in full-term neonates. The prevalence varies among different racial groups [1, 3, 4, 7, 12]. It is most commonly seen in Caucasians (37.8%) [2], than colored population. However, in a recent study conducted in Jordan, erythema toxicum neonatorum showed the highest prevalence rate of 68% in black-skinned population [13], which may suggest reasons other than racial factors. Erythema toxicum neonatorum has to be distinguished from other infective and noninfective pustular disorders in neonates [14].

**Figure 5:**
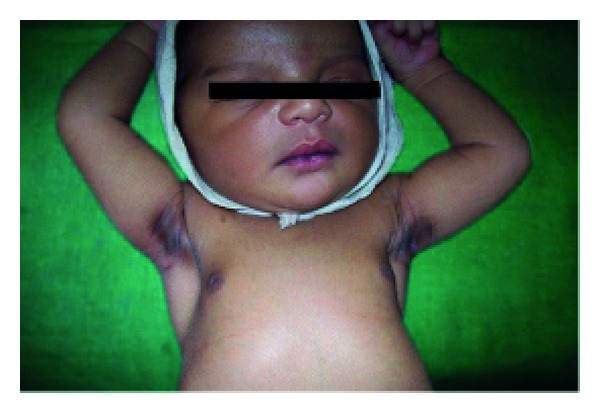
Axillary pigmentation.

**Figure 6:**
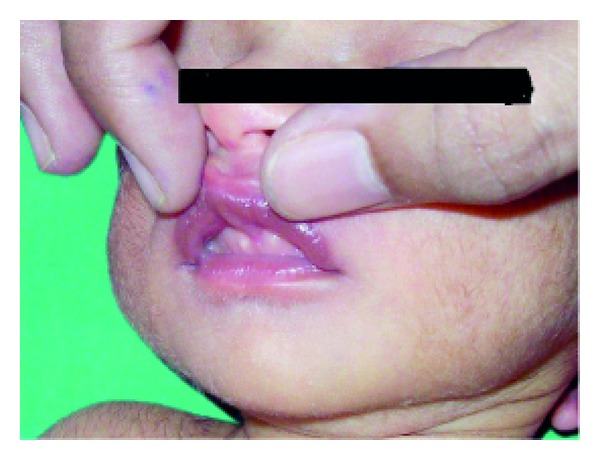
Bohn's nodules.

**Figure 7:**
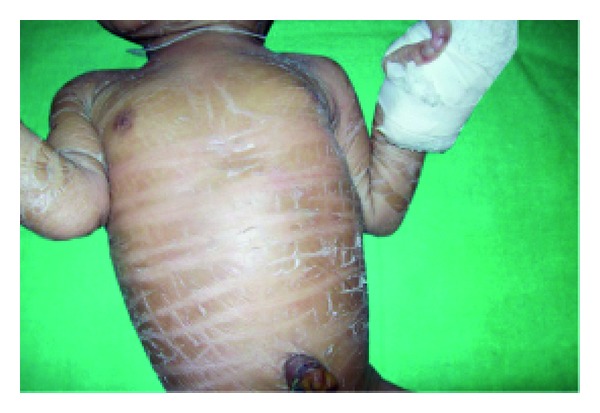
Scaling in ectodermal dysplasia.

**Figure 8:**
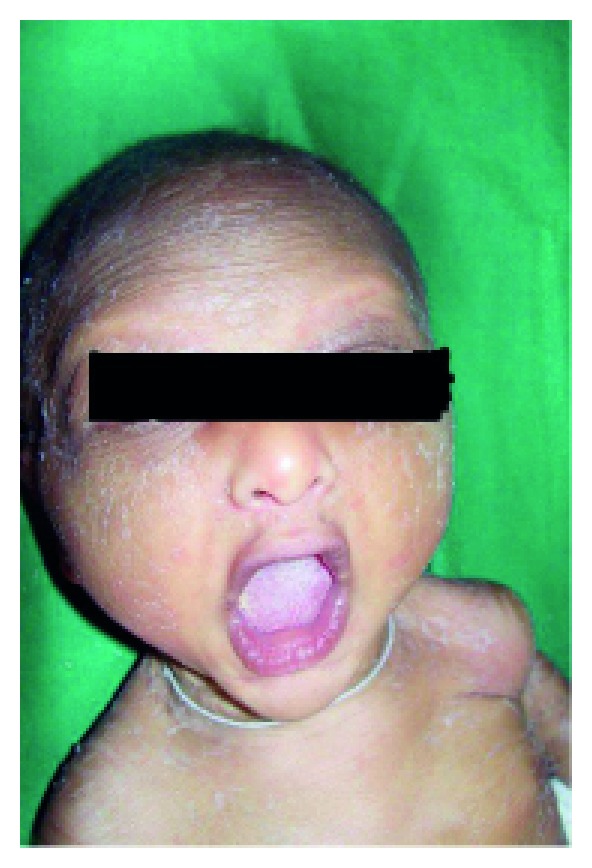
Senile changes in ectodermal dysplasia.

**Figure 9:**
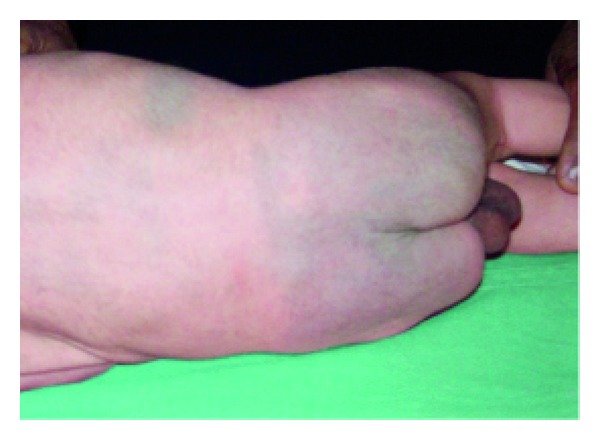
Mongolian spot over lumbosacral region.

**Figure 10:**
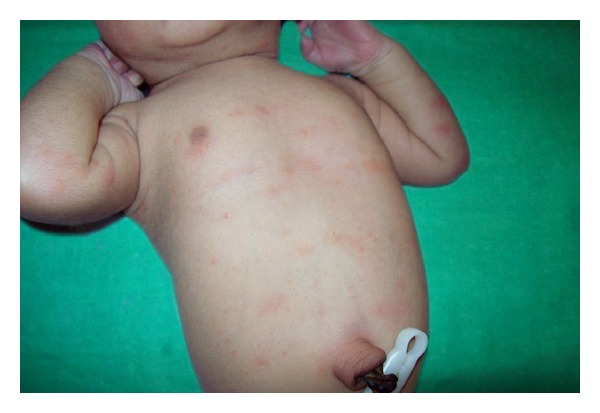
Erythema toxicum neonatorum.

**Figure 11:**
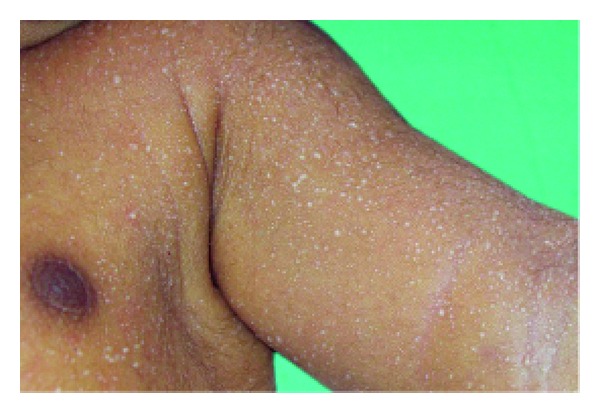
Miliaria crystallina.

**Figure 12:**
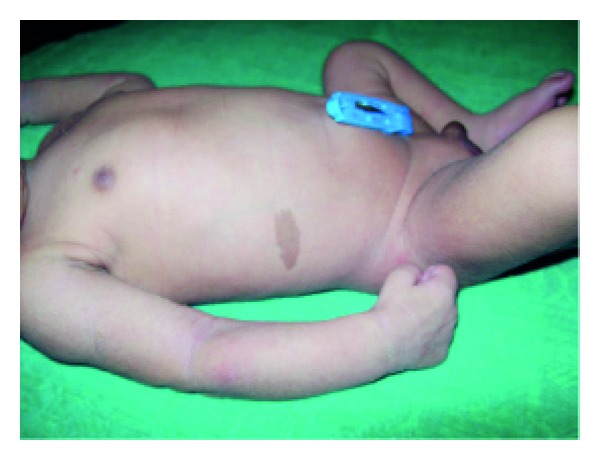
Café-au-lait macule.

**Figure 13:**
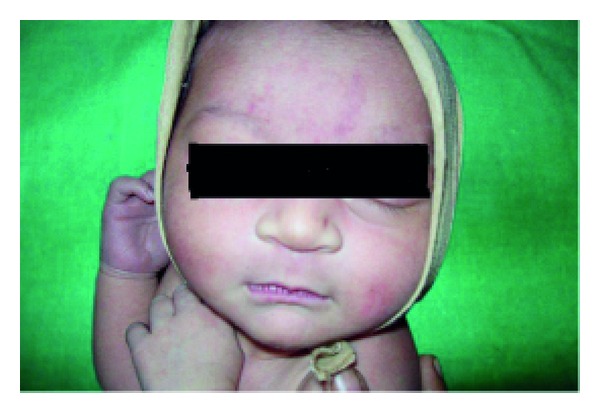
Salmon patch.

Among epidermal pigmentary changes, (Figure 5) linea nigra (44.5%) and knuckle pigmentation (57.9%) were most commonly seen. In a recent study by Pruksachatkunakom et al. [2], linea nigra was seen in 51.8% blacks and 5.0% Caucasians. It has been postulated to be a response to the maternal and placental hormones that enter the total circulation. Among these hormones, estrogen and progesterone have been reported to exert a melanocytic stimulating effect which also causes darkening of linea alba in pregnant women [2].

Physiological scaling, the most common finding, was seen in 105 (10.5%) neonates in the present study, compared to a study of Australian neonates, where the frequency of occurrence was 65% [11]. It was seen in 97 (92.38%) full-term neonates, 5 (4.76%) preterm, and 3 (2.88%) postterm neonates. Preterm infants showed desquamation in the present study, compared to other studies [1, 6], where desquamation was not seen in preterm neonates. The variation in prevalence is mainly because the duration of observation in our study was less than 4 weeks, the time when permeability barrier of preterm neonate undergoes maturation. However, in other studies the duration of observation was within 48 hrs of birth and premature infants do not show desquamation until 2-3 weeks of life. Vernix caseosa was seen in 7.7% of neonates. It was seen most commonly on 1st day of life. The prevalence of vernix caseosa has not been reported in previous studies.

Salmon patch was the most common vascular birthmark seen (20.7%). The prevalence of salmon patch varies in different studies. It is 22.3% in a Japanese study [7], 28.4% in an Indian study [9], 27.8% in a Taiwanese study [8], 18.78% in Jewish, and 19.97% in Arab neonates [15]. The most common site of occurrence in the present study was eyelids (90.82%). Female preponderances in vascular birthmarks were noted in Japanese survey [7]. In this study, males had higher prevalence (10.6%) than females (10.1%). However, this difference was not statistically significant (*P* = 0.6231).

The surveys of congenital melanocytic nevi in newborns showed a prevalence of 0.4 to 15.6%, with the highest percentage among nonwhitish babies [2]. An interesting study which adopted a comparative approach between Arabs and Jews in Israel, conducted by Kahana et al. [15], found that Arabs had greater number of melanocytic brown lesions (Mongolian spots, congenital melanocytic nevi, and café-au-lait macule) than Jews descending from European ancestry, but Jews descending from Asia and Africa had almost equal frequency of these melanocytic brown lesions as in Arabs. Congenital melanocytic nevi were seen in 19 (1.9%) newborns. The size of the nevi is important; nevi larger than 20 mm are considered to be giant type and are one of the precursors of melanoma [16]. Café-au-lait macules were seen in 13 (1.3%) neonates. One newborn had multiple café-au-lait macules and similar lesions were also seen in her mother and sibling. The prevalence of café-au-lait macule among Arabs is 0.48% and Jewish neonates is 0.11% [15].

Cutaneous signs of spinal dysraphism were seen in 135 (13.5%) neonates. Sacral dimple was seen in 128 (12.8%) neonates. The prevalence is higher than that in American neonates (1.4%) [2]. However, further investigations were not done for definitive diagnosis of spinal dysraphism. Meningomyelocele was seen in 5 (0.5%) neonates. None of these neonates showed any signs of neurological deficit except one neonate who was unable to move his limbs.

Vaginal tags and cowlicks hair pattern are two conditions which were not reported in previous observational studies on cutaneous manifestations in newborns. Vaginal tags were seen in 18 (1.8%) newborns. They are normal hymenal characteristic which can be seen in 59% of newborns [17]. Cowlicks hair is a normal frontal hair pattern seen in 7% of individuals and may have genetic contributions [18].

Anhidrotic ectodermal dysplasia was seen in one neonate who had loss of eyebrows, eyelashes with periorbital pigmentation. Skin biopsy done from hypothenar eminence of palm showed absent sweat glands [19].

## 5. Conclusion

Sebaceous gland hyperplasia, Epstein pearls, Mongolian spot, and erythema toxicum neonatorum were the commonest physiological and transient skin lesions seen in the study.

The study of newborn skin provides information about normal variants occurring in neonatal period. It is important to be aware of the fact that most of the skin lesions in the newborn are transient and require no therapy.

Therefore, it is necessary for those who provide neonatal care to differentiate physiological skin lesions from other more serious skin conditions which will help avoid unnecessary therapy to neonates. The parents can be assured of good prognosis of these skin manifestations.

## Figures and Tables

**Table 1:** Relationship of skin lesions with maternal-neonatal factors.

Maternal-neonatal factors	Total (*n*)	*Z*-test
(1) Sex		
Male	543	*P* = 0.0001
Female	457	(<0.05)

(2) Birth weight		
<2.50	576	*P* = 0.0000
>2.50	424	(<0.05)

(3) History of consanguinity		
Present	442	*P* = 0.0000
Absent	558	(<0.05)

(4) Parity		
Primi	450	*P* = 0.0000
Multi	550	(<0.05)

(5) Route of delivery		
Vaginal	688	*P* = 0.0000
Cesarean	312	(<0.05)

**Table 2:** Frequency of skin lesions in newborns.

Skin lesions	*N*	Percentage (%)
(1) *Physiological skin lesions *		
Vernix caseosa	77	7.7
Physiological scaling	105	10.5
Sebaceous gland hyperplasia	894	89.4
Milia	183	18.3
Epstein pearls	891	89.1
Hypertrichosis	353	35.3
Miniature puberty		
Hypertrophy of clitoris	23	2.3
Hypertrophy of mammary gland	75	7.5
Vaginal discharge	35	3.5
Pigmentary changes due to melanin		
(a) Epidermal		
Linea nigra	445	44.5
Pigmentation of pinna	496	49.6
Knuckle pigmentation	576	57.6
Genital pigmentation	202	20.2
Axillary pigmentation	256	25.6
(b) Dermal		
Mongolian spot	847	84.7
Pigmentation other than melanin		
Physiological jaundice	3	0.3
Color changes from vascular abnormalities		
Acrocyanosis	309	30.9
Harlequin color change	4	0.4
Cutis marmorata	38	3.8

(2) *Transient noninfective conditions *		
Erythema toxicum neonatorum	232	23.2
Miliaria crystallina	30	3
Eosinophilic pustulosis	1	0.1

(3) *Eczematous eruptions *		
Napkin dermatitis	1	0.1
Cradle cap	12	1.2

(4) *Birthmarks *		
Vascular		
Salmon patch	207	20.7
Haemangioma	2	0.2
Pigmentary		
Congenital melanocytic nevi	19	1.9
Café-au-lait macule	13	1.3

(5) *Developmental defects *		
Cutaneous signs of spinal dysraphism		
Sacral dimple	128	12.8
Acrochordons	5	0.5
Meningomyelocele	1	0.1
Dermoid cyst	1	0.1

(6) *Other developmental defects *		
Cleft lip	1	0.1
Cleft lip & palate	1	0.1
Umbilical granuloma	1	0.1
Supernumerary nipple	1	0.1
Accessory tragus	1	0.1
Adnexal polyp	1	0.1
Sacrococcygeal teratoma	1	0.1
Omphalocele	1	0.1
Perineal median raphe cyst	1	0.1

*Others *		
Anhidrotic ectodermal dysplasia	1	0.1
Congenital vitiligo	1	0.1
Phimosis	5	0.5
Cowlicks hair	2	0.2
Vaginal tags	18	1.8
Horizontal pigmented bands	2	0.2
Congenital hydrocele	1	0.1
Twin transfusion syndrome	1	0.1
HIV positive	1	0.1
Maternal varicella	2	0.2
Linear & whorled hypermelanosis	1	0.1

**Table 3:** Relationship of physiological skin lesion with sex.

Skin lesions	Males	Females	*Z*-test
Vernix caseosa	28	49	*P* = 0.0007
Physiological scaling	64	41	*P* = 0.0015
Epstein pearls	492	399	*P* = 0.0000
Hypertrichosis	194	159	*P* = 0.0084
Linea nigra	244	201	*P* = 0.0039
Genital pigmentation	113	89	*P* = 0.0169
Pigmentation of pinna	215	281	*P* = 0.0000
Knuckle pigmentation	327	252	*P* = 0.0000
Mongolian spot	467	380	*P* = 0.0000
Acrocyanosis	191	118	*P* = 0.0000

*P* < 0.05 statistically significant.

**Table 4:** Distribution of physiological skin lesions with respect to maturity.

Skin lesions	Full term		Preterm		Postterm	
	*N*	%	*N*	%	*N*	%
(1) Vernix caseosa	62	92.5	4	5.9	1	1.5
(2) Physiological scaling	97	92.38	5	4.76	3	2.85
(3) Sebaceous gland hyperplasia	789	88.25	86	9.61	18	2.01
(4) Milia	174	95.08	7	3.8	2	1.09
(5) Epstein pearls	776	87.09	88	9.8	27	3.03
(6) Hypertrichosis	293	83%	53	15.01	7	1.99
(7) Miniature puberty						
(a) Hypertrophy of clitoris	23	100				
(b) Hypertrophy of mammary glands	69	92	3	4	3	4
(c) Vaginal discharge	33	94.29	2	5.7		
(8) Pigmentary changes due to melanin						
(a) Epidermal						
Linea nigra	417	93.70	18	4.04	10	2.24
Pigmentation of pinna	128	86.29	56	11.29	12	2.41
Knuckle pigmentation	535	92.40	31	5.35	13	2.24
Genital pigmentation	183	90.60	12	5.94	7	3.46
Axillary pigmentation	241	94.14	8	3.13	7	2.73
(b) Dermal						
Mongolian spot	765	90.31	68	8.02	14	1.65
(9) Pigmentation other than melanin						
Physiological scaling	3	100				
(10) Color changes from vascular abnormalities						
Acrocyanosis	270	87.37	32	10.35	7	2.26
Harlequin color change	4	100				
Cutis marmorata	28	73.68	10	26.31		

**Table 5:** Distribution of birthmarks in neonates.

Birthmarks	Total	Percentage (%)
Salmon patch	207	20.7
Haemangioma	2	0.2
Congenital melanocytic nevi	19	1.9
Café-au-lait macule	13	1.3

**Table 6:** Prevalence of dermatoses in newborns in different racial groups.

Racial groups	EP (%)	SGH (%)	Milia (%)	MS (%)	ETN (%)
Australian neonate	56.0	48.0	36	25.5	34.8
Iranian neonate	70.2–88.27	43.7	7.5	71–81	11.1–54
Japanese neonate	—	—	—	81.5	40.8
Turkish neonate	—	31.8	1.4	13.2	30.9
Caucasians	35.7	46.3	—	62.8	37.8
Black neonate	29.1	28.2	—	86.6	11.6
Indian neonate	43.8–61	21.4	26.2	72–89	25
